# Impact of occupational death trauma on burnout among mental health professionals: the mediating role of secondary traumatic stress

**DOI:** 10.3389/fpsyt.2025.1543681

**Published:** 2025-06-03

**Authors:** Sun-Hee Park, Yun Hee Go, Heeran J. Cho, Myeong Sook Yoon

**Affiliations:** ^1^ Department of Social Welfare, Jeonbuk National University, Jeonju, Jeollabuk-do, Republic of Korea; ^2^ School of Glocal Studies, Kyungwoon University, Gumi, Gyeongsangbuk-do, Republic of Korea

**Keywords:** occupational death trauma, mental health professionals, secondary traumatic stress, burnout, vicarious trauma

## Abstract

**Introduction:**

In response to growing evidence regarding the psychological burden of client deaths on mental health professionals (MHPs), this study investigates how occupational death trauma influences burnout, particularly through the mediating role of secondary traumatic stress (STS).

**Methods:**

A structured online survey was administered to 224 MHPs working at mental health welfare centers across South Korea in June 2024. Multiple regression analysis and mediation analysis were performed using bootstrapping with 5,000 resamples in Stata/MP 18.0.

**Results and Discussion:**

Greater exposure to occupational death trauma was significantly associated with higher levels of burnout among MHPs (β = .515, p <.001). Occupational death trauma exerted a significant direct effect on burnout, and STS was found to partially mediate this relationship (β = .277, p <.001). Specifically, increased exposure to occupational death trauma elevated STS, which in turn intensified burnout among MHPs. These findings underscore both direct and indirect pathways through which occupational death trauma contributes to burnout, emphasizing the need for structured psychological interventions within the mental health workforce.

## 1 Introduction

Mental health professionals (MHPs) play an essential role in providing a diverse range of mental health services. These include preventive measures, promoting mental health, facilitating the deinstitutionalization of people with mental illnesses, preventing and intervening in suicidal crises, and supporting community adaptation and recovery for individuals with mental disorders (1–3). Among the diverse traumatic experiences encountered by MHPs, client death represents one of the most psychologically distressing events, often triggering acute emotional and professional disruption. Occupational death trauma refers to traumatic stress resulting from client death experiences encountered during professional duties. This includes both anticipated deaths (e.g., terminal illness) and sudden, unexpected deaths such as suicide, homicide, medical errors, or accidents. It can also encompass witnessing a client’s death or discovering a deceased client (4). Occupational death has been shown to severely affect both the personal lives and professional service delivery of mental health practitioners (5). Client deaths can provoke intense grief and affect MHPs’ emotional well-being and professional functioning over both the short and long term (6, 7).

Crucially, community MHPs are at a higher risk of exposure to trauma during their professional duties compared to those working in general mental health fields (8). According to a 2017 report by the National Center for Mental Health in South Korea (9), 80.6% of MHPs reported experiencing trauma, with 32% specifically exposed to occupational death trauma. Similarly, a study by Hyun and Kim (10) found that 35% of MHPs had encountered client deaths, and these individuals exhibited high levels of post-traumatic stress disorder (PTSD) symptoms.

Furthermore, client suicide often induces feelings of failure and incompetence among professionals, leading them to question their expertise and role, ultimately influencing changes in their clinical practices. MHPs are responsible for both suicide prevention and bereavement support, which inherently involves substantial occupational risks. Also, the death of a client by suicide has long been regarded as an occupational hazard for mental health workers (11). Maintaining a trusting relationship with clients and engaging in continuous interactions can lead to significant shock and loss, even when death is anticipated (12). According to a meta-analysis by Jupina et al. (13) on client suicide and its impact on health care professionals, 88% of the included studies reported that client suicide had an emotional impact on treating professionals, while 76% of the studies indicated a professional impact. MHPs, who frequently work with clients experiencing psychiatric disorders or trauma, are thus exposed to a higher frequency of distressing incidents compared to individuals in other occupations or the general population (14).

Notably, MHPs working in community mental health teams, who frequently establish close therapeutic bonds with clients, face substantial challenges in coping with client deaths. Specifically, 45% experienced prolonged anxiety, thoughts of resignation, or substance misuse (15). Moreover, both anticipated and unexpected client deaths have been found to result in grief and emotional distress among service providers (16, 17). These findings highlight the need for MHPs to acknowledge the emotional impact of client loss and to develop appropriate support and coping strategies in response.

For instance, when women firefighters are exposed to suicide during their careers, it has been associated with more severe psychiatric symptoms and an increased risk of suicide (18). A systematic review studying suicidal thoughts and behaviors among MHPs and first responders suggests a link between the psychological distress experienced in these occupations and higher rates of suicide (19).

In South Korea, the loss of a client—particularly through death—has been identified as one of the most distressing experiences of workplace trauma among MHPs (2). When a client dies during treatment, professionals experience personal grief; however, they often find themselves in situations where they have no formal means to express this mourning. This lack of formal acknowledgement can lead to doubts about their professionalism, especially when questions arise regarding the treatment process (12). Furthermore, professionals may experience feelings of helplessness and despair when confronted with client deaths, and the psychological burden of grief and distress can accumulate with repeated exposure to similar experiences (20). Client deaths and other life-threatening incidents can lead to heightened stress levels, manifesting as acute stress, PTSD, suicidal thoughts, and behaviors (19, 21, 22).

In addition to direct trauma, MHPs are also vulnerable to indirect psychological distress, commonly referred to as Secondary Traumatic Stress (STS), which may further intensify burnout. In addition to direct traumatic stress due to these experiences, the subsequent personal shock, professional responsibility, and guilt towards the bereaved can influence the onset of STS (14, 23, 24). STS refers to the emergence of PTSD symptoms following indirect exposure to trauma (25) and is recognized as a unique occupational risk among professionals working with trauma victims in mental health settings (25). In particular, STS is a reaction that can occur when MHPs become psychologically overwhelmed while helping clients, especially when they work with a strong sense of duty or commitment (26).

Many individuals are likely to encounter potentially traumatic events throughout their lives (27), with MHPs and helping professions such as doctors, nurses, social workers, and firefighters, reporting higher rates of occupational death trauma and STS (5). It is also predicted that professionals affected by STS are at a greater risk of making poor professional judgments compared to those who are not impacted (28, 29).

Recent studies have identified STS responses among MHPs who have experienced the death of clients. MHPs working in suicide prevention are frequently exposed to STS—not only due to the trauma of client suicides but also through ongoing support for suicide attempt survivors and the bereaved families of those who have died by suicide (8). In particular, MHPs who have experienced a client’s suicide report a range of psychological symptoms, including depression, sadness, anxiety, intrusive images and thoughts, and avoidance behaviors. These symptoms are indicative of a heightened vulnerability to STS in the aftermath of client death (30, 31). Leung et al. (32) conducted a systematic review examining the relationships among occupational death trauma, STS, vicarious trauma, and burnout in mental health workers, and reported that these relationships have statistically significant impacts.

Furthermore, correlations between STS and burnout have been identified among trauma-exposed human service providers, underscoring the emotional toll such work can take (26, 33, 34). These findings emphasize the need for greater institutional awareness and targeted interventions to support MHPs working in high-risk clinical environments.

Ongoing exposure to STS can undermine professionals’ competencies, increase doubts about their occupation, affect their self-esteem, and ultimately contribute to burnout (35). Burnout encompasses feelings of hopelessness, difficulties in effectively managing work, cynicism and detachment from the job, as well as a sense of inefficacy and lack of accomplishment (36). A comparative study of burnout prevalence among psychiatric nurses and public health nurses revealed significantly higher rates of burnout in psychiatric nurses (37, 38). Professionals in other trauma-related fields who have experienced client deaths have also been found to experience self-blame, shame, disruptions in occupational identity, and burnout during the course of their work (30, 39, 40).

This burnout negatively affects professionals’ job performance, and academic research has reported variations in the degree of burnout depending on job performance levels (41, 42). Additionally, job performance is a critical factor influencing burnout, and psychological counseling has been identified as a moderating factor in preventing burnout and alleviating job-related stress (43, 44). Moreover, burnout is not only an individual issue for field professionals but also leads to a decline in service quality, underscoring the importance of research on burnout among MHPs (45). Thus, trauma, STS, and burnout are frequently interconnected outcomes for MHPs due to their repeated exposure to client-related trauma (32). Previous studies have shown that the greater the case managers’ exposure to occupational death trauma, the more severe their burnout and the more negatively affected their professional performance (23, 46, 47). Furthermore, STS among MHPs has been found to significantly contribute to burnout (48, 49). Research also indicates that exposure to occupational death trauma impacts STS and is highly correlated with burnout (4, 35, 50). These findings, based on prior studies of professionals in human services, offer insights into the relationship between MHPs’ exposure to occupational death trauma, STS, and burnout. STS Theory posits that professionals in caregiving roles who are repeatedly exposed to others’ traumatic experiences may develop trauma symptoms themselves (51). This theory provides a framework for understanding the pathway through which STS leads to burnout. This theoretical framework is useful for explaining how repeated exposure to trauma among MHPs can lead to the accumulation of empathic fatigue and emotional exhaustion, ultimately resulting in decreased job satisfaction and burnout (33, 51). Moreover, STS can produce symptoms similar to those of PTSD, negatively affecting not only professionals’ psychological well-being but also the quality of care provided (26). Furthermore, repeated exposure to trauma warrants caution, as it may impair cognitive functioning and have detrimental effects on the formation of therapeutic relationships and overall clinical practice (52).

Despite the high frequency of client deaths in Korea’s mental health service settings, research on how these experiences affect MHPs, particularly through STS, remains scarce. In this context, it is crucial to examine not only the direct trauma that MHPs experience from client deaths but also the indirect psychological trauma, while also addressing the issue of burnout. Despite the frequent exposure of community-based MHPs to client deaths, little research has examined how such trauma leads to burnout via STS, particularly in the South Korean context. While MHPs are more likely to encounter client deaths than those in other practice settings, empirical studies on this issue remain scarce. In particular, empirical studies examining the psychological mechanisms—such as STS—that link occupational death trauma to burnout among Korean MHPs are notably lacking. While some studies have examined client loss and post-traumatic growth (2, 53) or experiences of client suicide (10, 54), there is a lack of research that specifically explores occupational death trauma and burnout. Consequently, the personal challenges faced by these professionals and the urgency of social and institutional support have not been adequately addressed.

**Figure 1:**
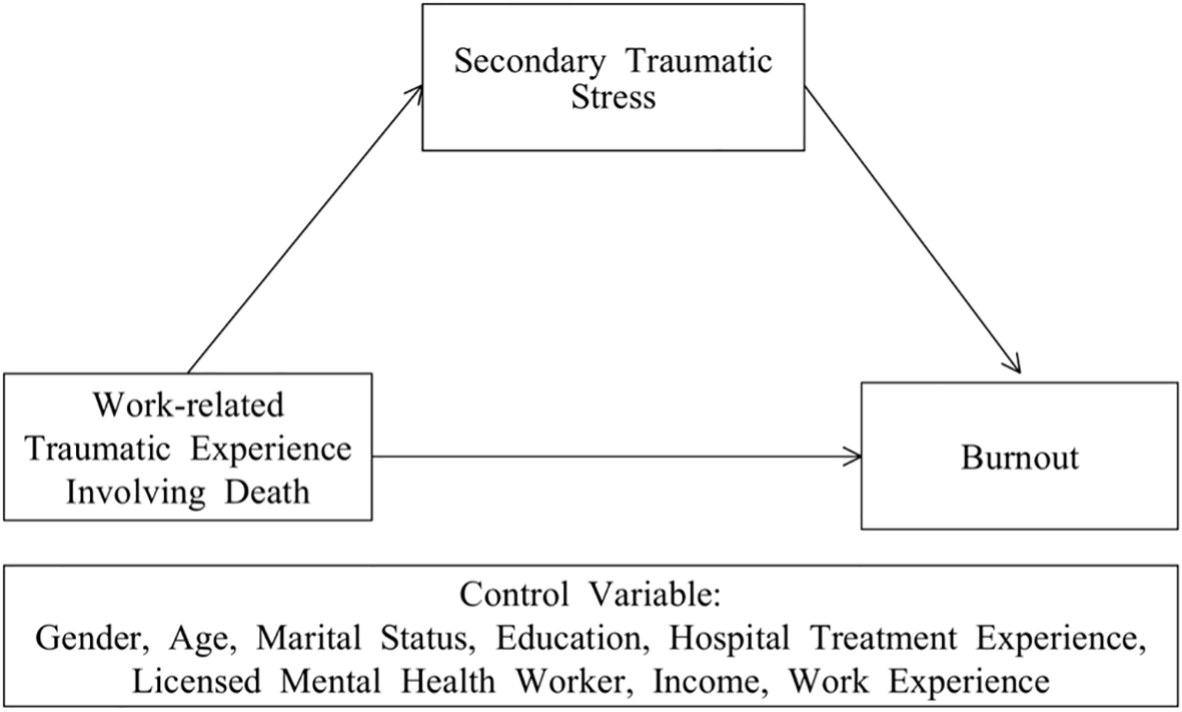
Conceptual framework of the mediation effect of STS on the relationship between occupational death trauma and burnout. H1 = Hypothesis 1: Occupational death trauma is expected to have a positive effect on burnout. H2 = Hypothesis 2: STS is hypothesized to mediate the relationship between occupational death trauma and burnout.

This study aims to analyze the effects of MHPs’ experiences of occupational death on burnout, investigate whether STS plays a mediating role in this relationship, and elucidate the pathways through which occupational death trauma affects MHPs. It also proposes strategies to mitigate burnout and enhance professional competencies. The conceptual framework guiding this study is illustrated in **Figure 1**. It depicts the hypothesized relationships between occupational death trauma, STS, and burnout among MHPs.

The hypotheses of this study are as follows:

Hypothesis 1 (H1): Occupational death trauma is expected to have a positive effect on burnout among MHPs.
Hypothesis 2 (H2): STS is hypothesized to mediate the relationship between occupational death trauma and burnout among MHPs.

## 2 Methods

### 2.1 Participants and data collection

In this study, participants were limited to MHPs working in community mental health public sectors, specifically those working in mental health welfare centers. A total of 224 community MHPs from mental health welfare centers in South Korea were surveyed using purposive sampling, which was deemed appropriate for targeting professionals with occupational death trauma. Additionally, the sample size was determined using multiple regression analysis, which considers the effects of multiple independent variables on the dependent variable. Using G*Power 3.1.9.7 software, the required sample size was calculated with an effect size of f² = 0.15 (moderate effect size), a significance level (α) of 0.05, and a statistical power (1-β) of 95%, incorporating predictor variables. The analysis resulted in a required sample size of 208 participants. Considering the potential for missing data and case exclusions, data were collected from a total of 224 MHPs. Since no errors or missing values were detected, all 224 responses were retained for the final analysis.

This study was conducted in accordance with the ethical principles outlined in the Helsinki Declaration, and prior approval was obtained from the Institutional Review Board (IRB) of Jeonbuk National University (JBNU 2024-06-033-001). Data collection transpired over a four-week period in June 2024, with promotional activities and the survey conducted online. The study’s purpose and survey instructions were thoroughly explained to the representatives of the institutions where the research participants were affiliated, either in person or via mobile communication. Participants were excluded from the survey if they had less than three months of clinical experience in the mental health field, had insufficient exposure to occupational death trauma, or had no experience receiving mental health counseling services.

Prior to participation, all respondents were informed that the survey would be conducted in a self-administered online format, allowing them to complete it independently. Participants were also informed that they had the right to withdraw from the study at any time without facing any disadvantages related to their participation. Although the survey was conducted anonymously, basic demographic information—including gender, age, and educational background—was collected. To ensure confidentiality, participants were explicitly informed that all collected data would be used exclusively for academic research purposes, in strict compliance with Articles 33 and 34 of the Korean Statistics Act and Articles 15 and 22 of the Personal Information Protection Act. Informed consent was obtained before data collection.

### 2.2 Measurement instruments

#### 2.2.1 Independent variable: occupational death trauma

To measure occupational death trauma, seven types of occupational death trauma constructed by Matzke (4) were utilized. The original questionnaire developed by Matzke (4) was translated into Korean and employed to measure the frequency of occupational death trauma.

The seven types of occupational death included unexpected medical accidents, suicides, homicides, accidental deaths, and deaths due to anticipated terminal illness. Participants were asked whether they had “witnessed a death” and “discovered the body” of clients. Questions regarding their experiences of occupational death during their time in the workplace were posed, with responses coded as ‘1 = yes’ for those who had the experience and ‘0 = no’ for those who did not. The possible score range for this measure was from 0 to 7, indicating the level of occupational death experience based on whether MHPs had experienced client deaths and how diverse those experiences were. A higher score indicates exposure to a greater variety of occupational death types, reflecting a higher level of trauma. In this study, the internal consistency of the occupational death trauma scale, measured by Cronbach’s alpha, was.645.

To evaluate the validity of the measurement tool, both convergent and discriminant validity were assessed. Convergent validity was evaluated using the Average Variance Extracted (AVE), which was calculated based on the communalities obtained from factor analysis. The AVE value, derived from the communalities of seven items, was 0.516, exceeding the commonly accepted threshold of 0.50 (55), thereby indicating an acceptable level of convergent validity. CR values exceeded 0.70, with most factor loadings above 0.60, indicating acceptable internal consistency. Discriminant validity was examined using the Fornell-Larcker criterion (56). According to this criterion, the square root of the AVE for each construct should be greater than the correlation between that construct and any other. In this study, two components (Component 1 and 2) were extracted. The correlation coefficient between the two components, derived the component transformation matrix, was 0.603, and its squared value was 0.364. Since this value was smaller than the AVE of each construct (0.516), the criterion for discriminant validity was satisfied. These results confirm the tool’s convergent and discriminant validity, supporting its psychometric adequacy.

#### 2.2.2 Mediating variable: STS

To measure STS, the Secondary Traumatic Stress Scale (STSS) developed by Bride et al. (24) was employed. The STSS consists of a total of 17 items grouped into three categories: “intrusion,” which includes 5 items related to the persistent, intrusive recollection of distressing events; “avoidance,” which consists of 7 items reflecting the desire to avoid reminders of the trauma; and “hyperarousal,” encompassing 5 items associated with chronic tension and symptoms such as insomnia. To minimize the influence of accumulated stress, participants were asked to reflect on their STS experiences over the past week. Each item is rated using a Likert scale ranging from ‘1 = not at all’ to ‘5 = very much.’ The scale has a minimum score of 17 and a maximum score of 85, with higher scores indicating a greater level of STS. In the original study by Bride et al. (24), the Cronbach’s alpha coefficient was reported to be.93, while in this study it was found to be.897.

#### 2.2.3 Dependent variable: burnout

For measuring burnout, the Maslach Burnout Inventory (MBI) developed by Maslach and Jackson (57) was used in this study. The MBI comprises a total of 22 items classified into three dimensions: emotional exhaustion (9 items), depersonalization (5 items), and reduced personal accomplishment (8 items). Originally, the MBI was scored on a scale from 0 to 6 points; however, in this study, a 5-point Likert scale was utilized, with responses ranging from ‘1 = not at all’ to ‘5 = very much,’ resulting in a total score ranging from 22 to 110. To enhance response consistency across instruments and reduce respondent fatigue, a 5-point Likert scale was employed. Higher scores indicate increased levels of burnout. Emotional exhaustion reflects feelings of mental fatigue, depersonalization signifies a growing emotional detachment and indifference towards others, and reduced personal accomplishment indicates negative feelings regarding one’s work performance and achievement. In this study, the Cronbach’s alpha coefficient for the MBI was found to be.846.

#### 2.2.4 Control variables

Gender, age, work experience, and professional competency (qualification status, degree) have been found to be related to burnout among various groups of professionals, such as social workers and mental health practitioners (37, 58). In this study, gender was coded as 1 = male and 0 = female. Education level was categorized as 1 = undergraduate degree and 0 = graduate degree or higher. Professional qualification was coded as 1 = Certified MHPs and 0 = Non certified-MHPs. Hospital treatment experience was recorded as ‘1’ for those with such experience and ‘0’ for those without. Age, monthly average salary, and current work experience were measured as continuous variables.

### 2.3 Data analysis

For the analysis, Stata/MP 18.0 and SPSS Process Macro version 4.2 were utilized. First, descriptive statistics and Pearson correlation analysis were conducted to examine the characteristics of the analytic sample and the relationships between key variables, including an assessment of multicollinearity. Second, a three-step regression analysis, as outlined by Baron and Kenny (59), was conducted to examine whether STS mediates the effect of occupational death trauma on burnout. Additionally, bootstrapping with 5,000 resamples was performed to verify the significance of the indirect effects, following the recommendations of Preacher & Hayes (60). Assumptions of normality, linearity, and homoscedasticity were verified and found to be satisfactory.

## 3 Results

### 3.1 General characteristics

The general characteristics of the study participants are presented in **Table 1**. The gender distribution included 73 males (32.6%) and 151 females (67.4%). The age breakdown revealed that 64 participants (28.6%) were in their 20s, 106 participants (47.3%) were in their 30s, and 54 participants (24.1%) were aged 40 and above, resulting in an average age of 34.24 years (SD = 7.032). Regarding educational background, 160 participants (71.4%) had graduated from university or below, while 64 participants (28.6%) had obtained a graduate degree or higher. In terms of hospital treatment experience, 102 participants (45.5%) had received treatment at least once, whereas 122 participants (54.5%) had never received treatment. With respect to professional qualification, 141 participants (62.9%) were licensed MHPs, while 83 participants (37.1%) were non-professionals.

**Table 1:** General characteristics of study participants (N = 224).

Category		Frequency (n)	Percentage (%)
Gender	Male	73	32.6
	Female	151	67.4
Age	20s	64	28.6
	30s	106	47.3
	40 and above	54	24.1
	M (SD)	34.24 (SD = 7.032)	
Education Level	Bachelor’s degree or lower	160	71.4
	Graduate degree or higher	64	28.6
Hospital Treatment Experience	Yes	102	45.5
	No	122	54.5
Certification	Certified MHP	141	62.9
	Non-certified MHP	83	37.1
Occupation	Mental Health Social Worker	110	49.1
	Mental Health Nurse	25	11.2
	Mental Health Clinical Psychologist	6	2.7
	Social Worker	59	26.3
	Nurse	17	7.6
	Psychologist	7	2.1
Monthly Average Salary	Less than $1,770.00	75	33.5
	$1,770.00 - $2,300.00	77	34.4
	$2,300.00 or more	72	32.1
	M (SD)	$ 1,890.77 (SD = 67.091)	
Work Experience (months)	Less than 2 years	122	54.4
	2 to 5 years	51	22.8
	More than 5 years	51	22.8
	M (SD)	40.23 months (SD = 46.118)		

MHP, Mental Health Professional; n, Number of Participants; %, Percentage; M, Mean; SD, Standard Deviation.

In terms of occupational classification, the largest group was mental health social workers (49.1%), followed by social workers (26.3%), mental health nurses (11.2%), and nurses (7.6%), mental health clinical psychologists (n = 6, 2.7%), and psychologists (n = 7, 2.1%). The monthly average salary revealed that 75 participants (33.5%) earned less than $1,770.00, 77 participants (34.4%) earned between $1,770.00 and $2,300.00, and 72 participants (32.1%) earned more than $2,300.00. Lastly, the experience in the current working institution was categorized as less than 2 years for 122 participants (54.4%), while 51 participants (22.8%) had between 2 and 5 years of experience, and 51 participants (22.8%) had over 5 years of experience.

### 3.2 Correlation and characteristics of key variables

In order to examine the correlations and multicollinearity among the key variables, a correlation analysis was conducted, and the results are presented in **Table 2**. The analysis indicated that the correlation coefficients among the independent variables were below.8. The correlation coefficient between occupational death trauma and burnout was relatively low (r = 0.397). This result suggests that occupational death trauma may influence burnout indirectly through delayed psychological responses, such as STS, rather than exerting a direct effect. Therefore, the direct association between occupational death trauma and burnout appears to be limited. Furthermore, this finding supports the notion that burnout is a complex phenomenon shaped by mediating psychological mechanisms rather than through a simple linear pathway.

**Table 2:** Correlation analysis and descriptive statistics of key variables (N=224).

Correlation	Occupational Death Trauma	STS	Burnout
Occupational Death Trauma	1		
STS	.499^***^	1	
Burnout	.397^***^	.577^***^	1
M	1.192	2.476	2.6
SD	1.399	.632	.452
Skewness	1.23	.293	.291
Kurtosis	4.327	2.908	3.144
Min	0	1.059	1.273
Max	7	4.412	4

M, Mean; SD, Standard Deviation; Min, Minimum; Max, Maximum; ^***^p<.001.

Furthermore, the variance inflation factor (VIF) was calculated to be 1.33, suggesting that multicollinearity is not an issue, as it does not exceed the threshold of 10. Moreover, skewness and kurtosis values were identified to have absolute values below 3 and 8, respectively, confirming that the data appears to exhibit a normal distribution. Among the occupational death trauma, suicide deaths were the most frequently reported, with 82 cases (36.2%), followed by predicted disease deaths with 80 cases (35.7%), and accidental deaths with 42 cases (18.0%).

### 3.3 Mediating effect of STS on the relationship between occupational death trauma and burnout

To investigate whether STS mediates the impact of occupational death trauma on burnout, a regression analysis was conducted, with results shown in **Table 3**. In the first step, STS was used as the dependent variable, incorporating the independent variables and control variables, which resulted in a statistically significant model. The analysis revealed that occupational death trauma had a positive effect on STS (β = .555, p <.001), with an explanatory power of 34.3% (F = 14.02, p <.001).

**Table 3:** The mediating role of STS in the relationship between occupational death trauma and burnout (N=224).

Variable		Step 1 (Independent → Mediator)			Step 2 (Independent → Dependent)			Step 3 (Independent → Mediator → Dependent)		
		B(SE)	t	β	B(SE)	t	β	B(SE)	t	β
Constants		2.537 (.241)	10.55^***^	·	2.949 (.173)	17.07^***^	·	2.17 (.193)	11.26^***^	·
Independent Variable	Occupational Death Trauma	.251 (.029)	8.67^***^	.555	.166 (.021)	8.01^***^	.515	.089 (.022)	4.09^***^	.277
Mediator Variable	STS	–	–	–	–	–	–	.307 (.044)	6.91^***^	.429
Control Variables	Gender (Male)	.010 (.076)	0.13	.008	.026 (.054)	.048	.026	.023 (.049)	.46	.024
	Age	.011 (.007)	1.64	.122	-.001 (.005)	-.22	-.017	-.004 (.004)	-1.02	-.069
	Education Level (Bachelor’s or below)	.133 (.085)	1.56	.095	.229 (.061)	3.75^***^	.230	.189 (.056)	3.39^**^	.189
	Hospital Treatment Experience (Yes)	.075 (.071)	1.05	.059	.024 (.051)	.46	.026	.001 (.047)	.02	.001
	Certified MHP	.372 (.096)	3.88^***^	.285	.139 (.069)	2.01^*^	.149	.024 (.065)	.038	.026
	Monthly Average Salary	-.004 (.001)	-4.33^***^	-.442	-.003 (.001)	-4.78^***^	-.490	-.002 (.001)	-3.10^**^	-.300
	Work Experience	.000 (.001)	0.45	.032	.003 (.001)	3.73^***^	.268	.002 (.001)	3.90^***^	.234
Model fit		F=14.02^***^ R^2^=.343 Adj R^2^=.318			F=13.62^***^ R^2^=.336 Adj R^2^=.312			F=20.05^***^ R^2^=.458 Adj R^2^=.435		

B, Coefficients; β, Standardized Coefficients; SE, Standard Error; ^*^p <.05, ^**^p <.01, ^***^p <.001.

In the second step, burnout was set as the dependent variable while including independent and control variables. The results indicated that occupational death trauma significantly affected burnout (β = .515, p <.001), with an explanatory power of 33.6% (F = 13.62, p <.001). In the third step, when burnout was treated as the dependent variable along with independent, mediating, and control variables, both occupational death trauma (β = .277, p <.001) and STS (β = .429, p <.001) exerted significant effects on burnout, producing an explanatory power of 45.8% (F = 20.05, p <.001). During this analysis, the β value for the occupational death trauma in the second step was found to be greater than that in the third step, indicating a partial mediation effect.

The results of testing whether the mediating effect of STS is statistically significant are presented in **Table 4**. The analysis indicated that the mediating effect of Occupational death trauma on burnout through STS was estimated to be.077. The Bootstrapped Upper Level Confidence Interval (ULCI) ranged from.046 to.111, which does not include 0. Furthermore, the direct effect of occupational death trauma on burnout was.089. The bootstrapping analysis yielded a 95% confidence interval of.046 to.133, which did not include zero, indicating that the direct effect was statistically significant. This suggests that, when considering the margin of error, the value produced is greater than 0, indicating statistical significance (61). This finding confirms the partial mediating effect of STS in the process by which occupational death trauma influences burnout.

**Table 4:** Verification of mediating effect significance.

Effect Type	Path	Effect	Boot SE	Boot LLCI	Boot ULCI
Direct effect	Occupational Death Trauma → Burnout	.089	.022	.046	.133
Indirect effect	Occupational Death Trauma → STS → Burnout	.077	.016	.047	.112

Boot SE, Bootstrapped Standard Error; Boot LLCI, Bootstrapped Lower Level Confidence Interval; Boot ULCI, Bootstrapped Upper Level Confidence Interval; Bootstrap samples = 5,000.

## 4 Discussion

This study emphasizes the pathways through which STS and burnout develop among MHPs in the context of client death. MHPs provide specialized services, including counseling, education, and training for individuals, groups, and families experiencing mental health challenges. The impact of occupational death trauma and STS on these professionals warrants greater attention, as such experiences intensify feelings of loss, raise doubts about professional competence, and significantly contribute to burnout. To examine this phenomenon, this study addressed two key research questions. First, does occupational death trauma influence burnout among MHPs? Second, does STS mediate the relationship between occupational death trauma and burnout? To investigate these questions, a survey was conducted among 224 community MHPs working at mental health welfare centers in South Korea. The collected data were analyzed using Stata/MP version 18.0.

### 4.1 Research findings and interpretation

The primary findings of this study are highlighted below. First, this study confirmed that as MHPs experience a higher incidence of occupational death trauma in occupational settings, their burnout levels increase. Burnout is a multidimensional construct consisting of complex subcomponents, including emotional exhaustion, depersonalization, and reduced personal accomplishment. Each subcomponent may respond differently based on trauma type and intensity, influencing job stress and psychological exhaustion.

This aligns with previous research that explains the phenomenon of MHPs experiencing occupational death and suffering from burnout (23, 47). Furthermore, the psychological impact experienced by professionals immediately following a traumatic event significantly contributes to burnout, even before the onset of STS. Following a client’s death, counselors often reflect on their professional role and responsibility, which can lead to feelings of doubt about job performance and contribute to burnout (12, 62). This finding supports previous studies indicating that job performance influences burnout (41–44). Specifically, the death of a client is shocking in itself, serving as a potential factor for questioning one’s professionalism and diminishing job commitment. Moreover, the death of a client entails additional responsibilities, such as providing bereavement counseling to that client’s family and friends. This prompts a critical consideration of the extent to which MHPs experience bereavement similar to the client’s family or close acquaintances? In this respect, it is important to remember that MHPs can sometimes find themselves in the role of clients who need help. The direct pathway between occupational death trauma and burnout suggests that traumatic events cause immediate and profound disruptions to an individual’s professional identity, job significance, and psychological resources. This finding indicates that experiencing occupational death trauma itself can directly contribute to emotional exhaustion, decreased job satisfaction, and avoidance behaviors (8). In practice, the psychological shock, grief, and sense of helplessness following exposure to occupational death trauma can intensify burnout levels regardless of the presence of STS. These findings emphasize the critical importance of psychological interventions immediately after a traumatic event and highlight the necessity of establishing support systems to directly address the immediate consequences of occupational death trauma in MHPs. The findings confirmed that occupational death trauma significantly predicted burnout (β = .515, p <.001), and STS partially mediated this relationship (indirect effect = .077, 95% CI [.047,.112]).

Second, it was confirmed that STS mediates the relationship between occupational death trauma and burnout. This finding aligns with existing research that indicates traumatic experiences related to death in the workplace influence STS and have a high correlation with burnout (4, 35, 50). Occupational death trauma negatively impacts an individual’s psychological resources and can indirectly contribute to burnout through STS (48). This suggests that the more frequently MHPs experience occupational death trauma, the higher their levels of burnout, with STS acting as an indirect pathway. In the relationship between occupational death trauma and burnout, STS is not merely a simple pathway but serves as a key mechanism through which trauma intensifies psychological distress, ultimately leading to burnout. The partial mediation effect confirms an indirect effect, demonstrating that as STS increases, burnout levels also tend to rise. Since exposure to death is an unavoidable aspect of MHPs’ work, recognizing and addressing STS at an early stage is crucial for preventing burnout and enhancing professional sustainability.

The finding that STS served as a partial mediator in the relationship between occupational death trauma and burnout suggests that STS functions as an important psychological mechanism linking occupational death trauma to burnout. However, it also indicates the existence of an independent pathway through which occupational death trauma can directly impact burnout without mediation by STS. This implies that the immediate emotional responses experienced by MHPs following trauma—such as psychological shock, guilt, role confusion, and loss of meaning in their work—may directly lead to emotional exhaustion or decreased motivation even prior to the development of STS symptoms.

Furthermore, STS is characterized by delayed onset symptoms that emerge after prolonged exposure to trauma, differentiating it from immediate acute stress responses. Considering these temporal characteristics and the mechanisms of manifestation, a full mediation model − which posits that occupational death trauma affects burnout solely through STS − may be regarded as having low theoretical and empirical validity. In other words, burnout symptoms may emerge solely from occupational death trauma even before STS has fully developed or been recognized. This provides theoretical support for the partial mediation effect confirmed in this study.

In particular, this highlights the importance of coping skills after experiencing a client’s death. It is crucial to identify signs of distress, such as STS, that may manifest before burnout fully develops. Professional interventions aimed at alleviating burnout enhance individual mental health, ultimately promoting well-being and a healthier life, while also playing a crucial role in improving work efficiency (41). In addition, this study included gender, age, education level, possession of qualifications, average salary, and work experience as control variables. These factors may have exerted potential confounding effects on the findings. For example, greater work experience or holding professional certifications may increase the likelihood of being exposed to high job-related stress, while lower levels of education could contribute to heightened vulnerability to burnout due to excessive workloads. The analysis showed that average salary was negatively associated with burnout, whereas work experience and the possession of qualifications were positively associated. Age appeared to function as a protective factor, reducing the risk of burnout, while lower educational attainment was linked to increased burnout levels. These results suggest that both individual and occupational characteristics play a substantial role in influencing burnout. Accordingly, these control variables should be carefully considered when interpreting the results, and future studies are encouraged to systematically examine their potential mediating and moderating effects.

A key contribution of this study is the expansion of the concept of occupational death trauma to include not only client deaths but also the experience of witnessing deaths as a traumatic event. This broader conceptualization enables a more comprehensive analysis of the various factors influencing occupational burnout. The examination of client deaths categorized into five types—unexpected medical accidents, suicides, homicides, sudden accidents, and anticipated disease-related incidents—and two types of witnessing—witnessing a death and witnessing a body—enables a more detailed analysis of factors affecting occupational burnout from various perspectives. While existing research primarily focused on tangible trauma from client assaults and verbal abuse, the exploration of trauma from client deaths facilitates an understanding of the multifaceted influence on burnout, extending to the psychological and emotional aspects of MHPs.

Moreover, this study is significant in that it analyzed the mediating effect of STS in the relationship between occupational death trauma and burnout among MHPs. STS manifests as a prolonged psychological impact, often leading to avoidance of trauma-related stimuli. Due to its subtle nature, it may remain unrecognized in daily life, making its effects particularly insidious. By investigating the mediating effect in the context of occupational death trauma and burnout, this research highlights the psychological and emotional challenges faced by MHPs. The findings can serve as foundational data that structurally confirms the sources of job-related stress and burnout among these professionals.

### 4.2 Recommendations

This study provides practical implications for MHPs and policymakers. In particular, MHPs who are repeatedly exposed to occupational death trauma could benefit directly from early screening for STS, systematic grief-processing protocols, and structured mental health recovery programs. These interventions contribute to the prevention of burnout and enhancement of professional sustainability, ultimately exerting a positive impact on the overall quality of mental health services.

The practical and policy recommendations based on the findings of this study are as follows. To enhance coping mechanisms for trauma related to client deaths and prevent burnout, national-level improvements should be made in developing professional recovery support manuals for managing such incidents in the mental health field. As this study confirms the significant impact of occupational death trauma on STS and burnout, it is essential to categorize psychosocial trauma alongside other forms of trauma, such as physical assault and verbal abuse, and establish appropriate guidelines. Currently, in the South Korean community service system, professionals often return to work without adequate mourning processes and are left to handle incidents and related legal procedures independently (63). This situation can compel professionals to shoulder the entire burden themselves. Even though guidelines may exist within organizations, their practical implementation often falls short due to real-world conditions. Therefore, specific alternatives that encompass case reporting and legal responses related to client deaths, coupled with the responsibilities of the affiliated institutions, should be supported by national guidelines.

Second, as the stress resulting from trauma among MHPs is often internalized rather than outwardly expressed, regular assessments and structured recovery support programs should be implemented for high-risk groups. Regular screening for workers is essential to identify high-risk groups and provide self-care opportunities. Self-care is recognized as a crucial factor in reducing stress and preventing burnout (64). Given the findings of this study, which confirmed a relationship between occupational death trauma, STS, and burnout, it is necessary to conduct regular assessments using checklists to prevent issues that could impair the professional capabilities of these specialists due to burnout. Additionally, applying assessment scales that correspond to each trauma incident for regular evaluations will help prevent the deterioration of professionals’ capacities. While there are regular capacity-building trainings centered around MHPs, these tend to focus predominantly on theoretical education and training, lacking content aimed at recovery support for psychological trauma. Particularly, while specialized knowledge is essential for MHPs, their ability to concentrate on work can be significantly hindered if they do not possess psychological stability. Thus, there is a need for proactive recovery support programs, especially for high-risk groups, rather than merely relying on assessments.

MHPs should also be recognized as survivors who experience the loss of clients, similar to bereaved families, and therefore require individual counseling and psychological therapy to mitigate burnout (43, 44). In South Korea, public officials exposed to high-risk traumatic events are provided with leave for psychological stability and financial support for mental health treatment under the National Civil Servant Service Regulations. However, unlike public officials who benefit from state-level psychological support systems, MHPs predominantly work in the private sector, where institutionalized psychological recovery and job support mechanisms following occupational death trauma remain inadequate. This lack of support poses a risk of repeated occupational death trauma, which can lead to increased burnout and diminished job performance. Ultimately, this may result in a decline in service quality and gaps in client care (43, 64). Therefore, the Ministry of Health and Welfare must urgently establish systematic workforce management and develop an integrated support system for MHPs. To ensure equitable access to psychological support among high-risk occupational groups, legally institutionalizing comprehensive mental health support and management systems for MHPs and other high-risk workers is imperative. Such measures would not only enhance individual recovery but also contribute to the sustainability of mental health services and the overall improvement of public health quality (36).

## 5 Limitation

While this study offers valuable insights into the occupational death trauma of MHPs related to client deaths, certain aspects warrant consideration for future research. Firstly, this study did not differentiate the psychological impact based on the various types of client deaths, could limit the transferability of these findings. Additionally, the sample may be concentrated within specific institutions or regions, potentially limiting the representativeness of the results. Unmeasured confounders, such as prior personal occupational death trauma among participants, were not controlled for and could have introduced bias. Furthermore, this study focused on total scale scores for STS and burnout without analyzing sub-factors, which could provide a more nuanced understanding of the relationships among variables.

Future research should consider longitudinal designs to address potential reciprocal causality and enhance the generalizability of the findings across different cultural contexts. Expanding the research to include a broader range of organizations and analyzing various forms of client deaths could provide deeper insights. Investigating the differences between personal occupational death trauma of MHPs and their occupational death trauma would also be beneficial. Incorporating objective data analysis on case reporting procedures, media interactions, and legal disputes following a client’s death can further enrich the understanding of this field. Additionally, exploring how control variables such as education level, income, and work experience may exert moderating effects could offer valuable perspectives.

## 6 Conclusions

Due to the unique nature of the mental health field, it is crucial for MHPs to manage stress and prevent burnout effectively. As their primary responsibilities involve direct client interactions through counseling, their psychological and emotional well-being directly influences their professional competencies and overall quality of life. The findings indicate that occupational death traumas significantly affect burnout, with STS identified as a partial mediator. This suggests that in the aftermath of occupational death trauma, MHPs require structured grief-processing mechanisms, along with specialized training and recovery support programs that directly address STS-related distress. To mitigate the risk of burnout, policies should establish structured psychological recovery frameworks, including mandatory trauma debriefing sessions and subsidized counseling services for MHPs following client deaths. This study offers theoretical and empirical insights into STS-related burnout among MHPs and empirically verifying the impact of occupational death trauma on burnout, as well as the mediating effect of STS. Through this, the study underscores the importance of managing and preventing STS and proposes specific intervention strategies and policy implications for mitigating burnout among MHPs. Future institutional efforts should prioritize early intervention protocols and long-term mental health strategies to safeguard MHPs from the cumulative impact of occupational death trauma.

## Data Availability

The raw data supporting the conclusions of this article will be made available by the authors, without undue reservation.
